# Combined frontal and parietal P300 amplitudes indicate compensated cognitive processing across the lifespan

**DOI:** 10.3389/fnagi.2014.00294

**Published:** 2014-10-24

**Authors:** Rik van Dinteren, Martijn Arns, Marijtje L. A. Jongsma, Roy P. C. Kessels

**Affiliations:** ^1^Research Institute BrainclinicsNijmegen, Netherlands; ^2^Donders Institute for Brain, Cognition and Behavior, Radboud University NijmegenNijmegen, Netherlands; ^3^Department of Experimental Psychology, Utrecht UniversityUtrecht, Netherlands; ^4^Donders Institute for Brain, Cognition and Behavior, Radboud University Medical CenterNijmegen, Netherlands; ^5^Behavioural Science Institute, Radboud University NijmegenNijmegen, Netherlands; ^6^Department of Medical Psychology, Radboud University Medical CenterNijmegen, Netherlands

**Keywords:** event-related potential (ERP), P300, P3, oddball, aging, CRUNCH, neural development trajectory, frontal compensation

## Abstract

In the present study the frontal and parietal P300, elicited in an auditory oddball paradigm were investigated in a large sample of healthy participants (*N* = 1572), aged 6–87. According to the concepts of the compensation-related utilization of neural circuits hypothesis (CRUNCH) it was hypothesized that the developmental trajectories of the frontal P300 would reach a maximum in amplitude at an older age than the amplitude of the parietal P300 amplitude. In addition, the amplitude of the frontal P300 was expected to increase with aging in adulthood in contrast to a decline in amplitude of the parietal P300 amplitude. Using curve-fitting methods, a comparison was made between the developmental trajectories of the amplitudes of the frontal and parietal P300. It was found that the developmental trajectories of frontal and parietal P300 amplitudes differed significantly across the lifespan. During adulthood, the amplitude of the parietal P300 declines with age, whereas both the frontal P300 amplitude and behavioral performance remain unaffected. A lifespan trajectory of combined frontal and parietal P300 amplitudes was found to closely resemble the lifespan trajectory of behavioral performance. Our results can be understood within the concepts of CRUNCH. That is, to compensate for declining neural resources, older participants recruit additional neural resources of prefrontal origin and consequently preserve a stable behavioral performance. Though, a direct relation between amplitude of the frontal P300 and compensatory mechanisms cannot yet be claimed.

## Introduction

### The P300 complex

**Figure 1:**
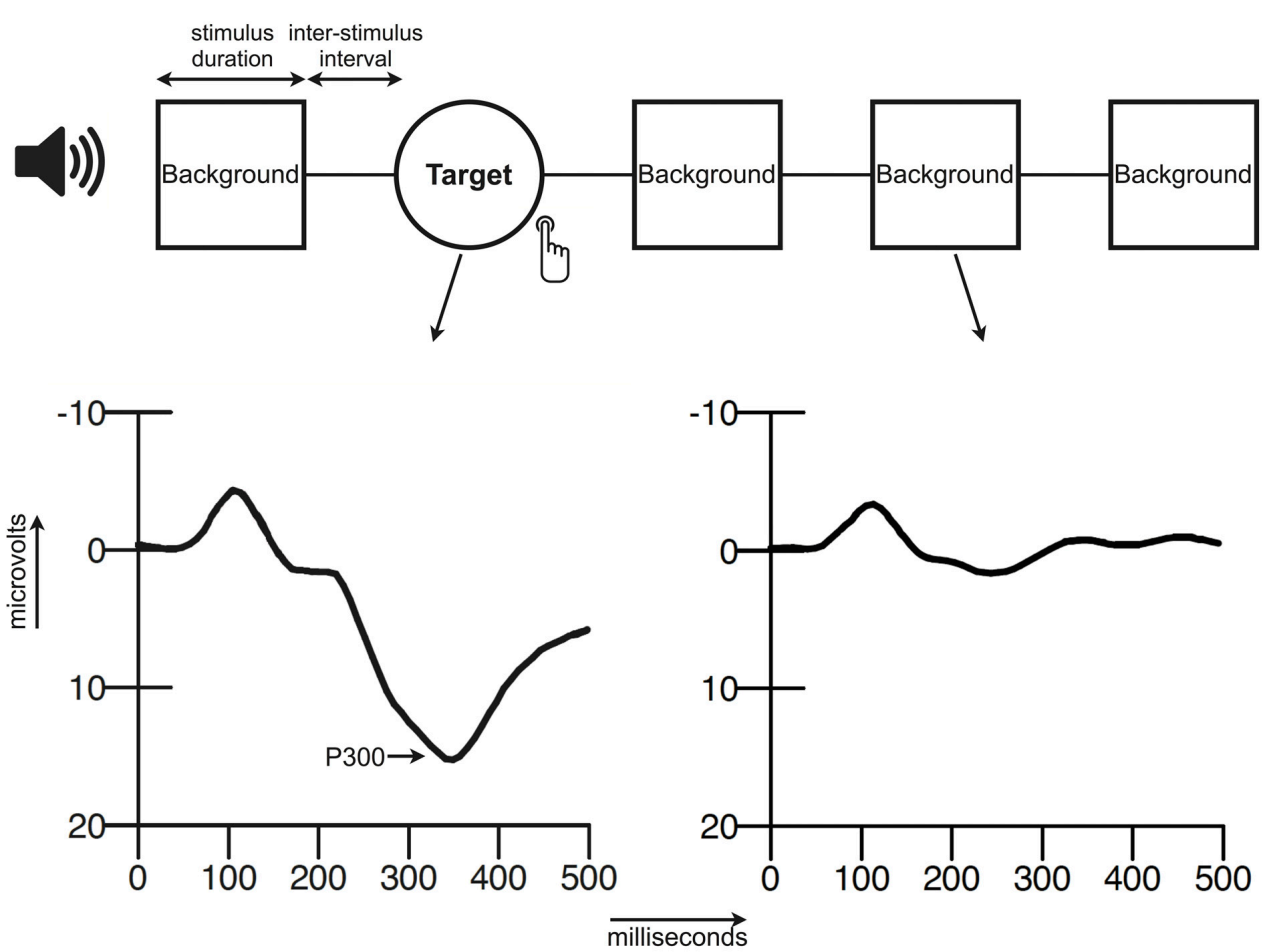
**Schematic overview of the oddball paradigm and an ERP**.

The event-related potential (ERP) is a waveform that is commonly determined by averaging brain activity in the electroencephalogram (EEG) time-locked to a specific event, for instance an auditory stimulus. The ERP consists of a series of components that can be distinguished based on their latency (ms), polarity (positive/negative), amplitude (μV), and scalp distribution. The P300 complex of the ERP is a large positive waveform that reaches its peak amplitude at approximately 300 milliseconds after stimulus presentation (Sutton et al., 1965). An advantage of the P300 with respect to the other components of the ERP is its relatively large size, which makes it easy to detect. The P300 that is elicited in the auditory oddball consists of neural activity originating from presumably the prefrontal cortex, the temporoparietal junction, the primary auditory cortex and possibly more sources (Friedman, 2003). In the oddball paradigm a participant is presented with two (or more when distracter stimuli are included) different stimuli that have to be discriminated. The stimuli can differ for instance in pitch, loudness, or duration, and are commonly presented with two different probabilities (e.g., 20 vs. 80%). The infrequent stimulus is generally designated as the target stimulus and participants are instructed to respond to this target stimulus, e.g., by a button press or by silent counting (Ritter and Vaughan, 1969; Polich, 1996, 2004). For a schematic overview of an ERP elicited within an oddball paradigm see Figure 1. The oddball is a simple task that can be executed by very young children, adults and the elderly. This is an important advantage in aging studies that, like this paper, investigate a broad age range.

Besides numerous studies demonstrating the traditional parietal P300, which is associated with responding to infrequent target stimuli, another P300 peak has been reported. This peak has a slightly faster latency and a more anteriorly distributed topography, and is most pronounced in oddball paradigms that include a third infrequent non-target stimulus. This peak has been labeled P3a (Squires et al., 1975; Snyder and Hillyard, 1976; Courchesne et al., 1984; Comerchero and Polich, 1999; Patel and Azzam, 2005), and consequently the traditional P300 as described in the previous paragraph has also been labeled P3b. In a standard two-tone oddball paradigm the P3a cannot be identified easily and perhaps, the P3a is super-positioned on the more pronounced P3b wave—specifically in the frontal cortex—in participants that do not generate a clear P3a peak. Because in this paper the focus lies on information processing in general and a two-tone auditory oddball is used, whenever the term “P300” is used it refers to this entire P300 complex. The terms frontal and parietal are used to refer to the location where the P300 is measured.

### The P300 amplitude as an index for cognitive aging

The P300 has been associated with various cognitive processes like attention, working memory, and executive function. One of the most prominent hypotheses linking the P300 amplitude to cognitive functioning is the context-updating hypothesis. This hypothesis states that the mental model a participant has of his/her current environment (context) is evaluated and updated when a relevant and deviant stimulus is introduced into the environment (Donchin, 1981). However, the context-updating hypothesis is only one of several hypotheses aimed at explaining the underlying cognitive mechanisms of the P300. Others are for example, the stimulus-evaluation hypothesis (Duncan-Johnson and Kopell, 1981), the context-closure hypothesis (Verleger, 1988) and the attentional resource-allocation model (Polich, 2007). Recently, a robust centro-parietal positivity in an averaged ERP signal has been related to decision-making processes. This positivity showed a strong resemblance to the traditional P300 and might revitalize the idea of the P300 reflecting a decision making process (Hillyard and Kutas, 1983; Nieuwenhuis et al., 2005; O'Connell et al., 2012b; Kelly and O'Connell, 2013). Another recent study hypothesized that the P300 is mainly indicating activation of rare stimulus-response links. Additionally, for smaller parts the P300 also reflects components of pure stimulus-related and pure response-related processes (Verleger et al., 2014). Although the P300 remains fairly elusive with respect to the involved cognitive processes, there is broad consensus that the P300 indexes aspects of cognitive information processing. For example, the P300 amplitude can be seen as an index for the amount of cognitive resources that a participant allocates in a cognitive task (Polich, 2007). Thus, with an increase in mental effort, P300 amplitude will also increase. A decline in P300 amplitude with increasing task difficulty has also been reported (Kim et al., 2008; Choi et al., 2010). P300 amplitudes are smaller when task difficulty is manipulated by increasing the similarity between the target and background stimulus (e.g., reducing the difference in pitch), creating uncertainty whether a stimulus is a target or not. Thus, in a difficult task, P300 amplitude increases when a participant devotes more effort to it, but decreases when the participant is less certain of the stimulus category (Luck, 2005, p. 44).

In our previous study, we modeled the developmental trajectory of the parietal P300 amplitude measured at Pz across the lifespan in a large cross-sectional sample (*N* = 1572). Furthermore, we performed a systematic review and meta-analysis on lifespan changes in the parietal P300 amplitude that showed similar results as were found in the cross-sectional sample. The amplitude of the parietal P300 was found to increase during childhood to reach a maximum around the age of 21, after which it gradually decreased, in line with evidence on cognitive decline from early adulthood to old age. Despite the observed decline in P300 amplitude during adulthood, behavioral performance remained relatively stable until into old age. In this earlier study we hypothesized that there might be neural processes in older age that compensate for this parietal P300 amplitude decline (van Dinteren et al., 2014).

### Compensation from prefrontal regions

Compensatory brain activity in older age has been described in several ways. One hypothesis, the Compensation-Related Utilization of Neural Circuits Hypothesis, or “CRUNCH,” states that the aging brain recruits compensatory neural resources when solving a task in order to maintain the achieved output (i.e., the behavioral performance) at a level that is equivalent to that of a younger brain. Firstly, the aging brain may increase activity in a certain neural network to compensate for declining processing efficiency in that same network. In addition, compensation might be achieved by increased activity in other, yet connected networks. Thus, increasing the activity in a certain or alternative network may reflect compensation for reduced neural processing. Only once the limits of increased activation in a certain network and recruiting alternative neural resources are reached, task performance will decline (Reuter-Lorenz and Cappell, 2008; Cappell et al., 2010; Daffner et al., 2011).

Other relations between compensation and frontal brain activity have been found as well. Cabeza et al. (2002) compared high vs. low performing old adults and demonstrated that the older low performers used neural networks similar to young participants (unilateral frontal activation), but in an inefficient manner. The old high performers compensated through a reorganization of neurocognitive networks demonstrated by bilateral frontal activation. This model was called Hemispheric Asymmetry Reduction in Old adults or HAROLD (Cabeza et al., 2002) (also see Berlingeri et al., 2013, for a comparison of the HAROLD model vs. the CRUNCH model). A posterior-to-anterior age-related shift in the recruitment of neural resources (Posterior-Anterior Shift in Aging or PASA) has been suggested to reflect compensation for age-related changes in posterior regions (Davis et al., 2008). Recently this research group demonstrated a negative correlation between white-matter deficits and over-activation in the prefrontal cortex and medial temporal lobes in older adults that showed successful behavioral performance in executive function and memory tasks (Daselaar et al., 2013). Geerligs et al. (2012) observed an increased relation between inhibition inefficiency and phase locking in the higher beta band between frontal and occipito-parietal regions. High-performing older patients showed more phase locking and they suggested that this represented a compensation mechanism leading to more effective inhibition. Li et al. (2013) found that, during attention, older adults increasingly recruited neural networks that were more frontally distributed. They suggested this might be compensatory to age-related decreases in inhibition capacities. A recently published study using fMRI during a working memory task, demonstrated brain activations in the left dorsolateral prefrontal cortex related to aging. The nature of this relation was dependent on task load. At low task demands, activation in this region increased with aging, whereas at higher task demands (and when participants reached their capacity limits), activation in this region decreased with aging. In the low task demand condition increased frontal activation was related to a decrease in behavioral performance, which was not in line with the concepts of CRUNCH. Possibly, this frontal activation in this task condition may reflect inefficient neural processing or failed compensation (Toepper et al., 2014).

In all, when compensatory activity is based on recruitment of alternative neural circuits, it has been proposed that these alternative neural resources are predominantly recruited from prefrontal cortices. Recruiting compensatory frontal neural circuits seems also in line with the often reported anterior shift in P300 topography with aging (Friedman et al., 1997; West et al., 2010; O'Connell et al., 2012a; Li et al., 2013). While it remains under debate whether increased frontal activation related to aging is either reflecting compensatory processes or inefficient cognitive processing (Friedman, 2003; Grady, 2012), it may well be that the same neurocognitive mechanism underlies both. That is, when inhibitory abilities decrease with aging, increasing frontal activity may reflect failed or successful compensatory mechanisms in respectively low and high performing older participants. As Grady (2012) stated in a recent review, compensation may come in three forms: (1) Increased brain activation without an effect on behavioral performance (attempted compensation), (2) increased brain activity associated with improved behavioral performance (successful compensation), or (3) increased brain activity associated with a decline in behavioral performance (unsuccessful compensation).

### The present study

Assuming the P300 amplitude reflects cognitive functioning in general, it might be suitable as an index for neurocognitive aging. This is supported by findings from others, for instance Walhovd and Fjell (2001), who demonstrated that the P300, measured at Cz and Pz, showed strong correlations with age. Interestingly, this correlation was not observed for the P300 amplitude measured at Fz. Possibly, cognitive processing reflected by a frontal P300 might be different from the parietal P300. One of the explanations for this apparent discrepancy might lie in the concept of frontal compensatory brain activity.

Since the ERP reflects cumulative neural activation related to multiple processes involved in stimulus processing, these parallel processes (i.e., the underlying ERP components) are likely to have overlapping post-stimulus latencies (Luck, 2005; Luck and Kappenman, 2013). Congruently, the P300 has been found to consist of multiple underlying independent components or sources. For instance, using independent component analysis (ICA), Onton et al. (2006) found three independent components that project to posterior cortical areas, and one projecting to the frontal cortical areas. In addition, intracranial studies, lesion studies and fMRI-EEG studies (see the review by Linden, 2005) and analyses with Low Resolution Electromagnetic Tomography (LORETA) (Mulert et al., 2004) point toward multiple neural generators of the P300. Possibly, prefrontal compensatory mechanisms may weigh heavier on the P300 measured at an anterior location compared to the P300 measured at a posterior location. If the P300 measured at Fz consists of another source mix (in other words reflecting a different mixture of cognitive processes) than the P300 at Pz, and this source mix changes with aging, differences in developmental trajectories are to be expected.

In this study we aim to further test this hypothesis and firstly replicate the finding from Walhovd and Fjell (2001) that the frontal P300 does not decrease with age. Secondly, we compared the frontal and parietal P300 amplitude developmental trajectories. Thirdly, we investigated whether the combined parietal and frontal P300 lifespan trajectory is more clearly associated with the behavioral performance trajectory in an oddball task, as compared to separate frontal or parietal P300 lifespan trajectories. More precisely, it is expected that the amplitude of the frontal P300 will be lower than the amplitude of the parietal P300 in children. Since frontal brain structures mature relatively late (Thatcher et al., 1987; Gogtay et al., 2004), neural activation originating from frontal structures is lower at young age. In our earlier study, we observed a decrease in amplitude of the parietal P300 with aging (van Dinteren et al., 2014). Based on CRUNCH, we expect that, unlike the parietal P300, the frontal P300 will steadily increase in magnitude with advanced aging, reflecting an increase in recruitment of compensatory frontal neural circuits that predominantly weigh on the frontal P300. In short, the developmental amplitude trajectories of the frontal and parietal P300 are expected to be markedly different with aging. This would also indicate that a P300 measured only at a posterior site would be insufficient to index cognitive aging. A lifespan trajectory of combined frontal and parietal P300 amplitudes, reflecting compensated neural activity, is expected to be related more closely to behavioral performance. As the P300 latency may index information-processing speed (Fjell et al., 2009; van Dinteren et al., 2014), latencies of both the frontal and parietal P300 are expected to increase with aging reflecting an overall decline in processing speed.

## Methods

### Participants

Data of individuals in the age range of 6–87 were extracted from the Brain Resource International Database (BRID). This database contains data from multiple laboratories (New York, Rhode Island, Nijmegen, London, Adelaide, and Sydney) that have been acquired using standardized data acquisition techniques (identical amplifiers, standardization of other hardware, audio calibration, paradigm details, software acquisition, and task instructions). Inter-lab reliability and test-retest reliability measures are high and have been reported elsewhere (Williams et al., 2005; Clark et al., 2006; Paul et al., 2007). Database exclusion criteria included a personal or family history of mental illness, brain injury, neurological disorder, serious medical condition, drug/alcohol addiction, first-degree relative with bipolar disorder, schizophrenia, or genetic disorder.

The sample consisted of 1572 participants (786 males; mean age = 27.17 ± 19.16). Age distributions are listed in Table 1. For every age (e.g., 6, 7, 8,…) sex distributions were 50/50%. Education scores varied from 1 to the maximum score of 18 years of education (mean = 11 ± 4). Participants were required to refrain from caffeine and smoking (2 h) and alcohol (6 h) prior to testing. All participants gave written informed consent. This sample was identical to the sample used in our previous study (van Dinteren et al., 2014).

**Table 1:** **Age distributions**.

**Age group**	***N***
6–9	118
10–19	704
20–29	246
30–39	126
40–49	100
50–59	116
60–69	96
70–79	62
80–83	4

### Electroencephalographic data acquisition

EEG and ERP recordings were performed using a standardized methodology and platform (Brain Resource Ltd., Australia). Participants were seated in a sound and light attenuated room, controlled at an ambient temperature of 22°C. EEG data were acquired from 26 channels: Fp1, Fp2, F7, F3, Fz, F4, F8, FC3, FCz, FC4, T3, C3, Cz, C4, T4, CP3, CPz, CP4, T5, P3, Pz, P4, T6, O1, Oz, and O2 using a Compumedics Quick-Cap with sintered Ag/AgCl electrodes and Neuroscan NuAmps DC amplifier with a 100 Hz low-pass filter (range is DC-100 Hz). Data were offline referenced to averaged mastoids with a ground at AFz. Horizontal eye movements were recorded with electrodes placed 1.5 cm lateral to the outer canthus of each eye (bipolar). Vertical eye movements were recorded with electrodes placed 3 mm above the middle of the left eyebrow and 1.5 cm below the middle of the left bottom eyelid. Skin resistance was aimed at <5 kOhms for all electrodes. A continuous acquisition system was employed and EEG data were EOG corrected offline (Gratton et al., 1983). The sampling rate of all channels was 500 Hz.

### ERP scoring

Conventional ERP averages were obtained from Fz and Pz recording sites for target stimuli. Only stimuli with a correct target response were included in the target average. Before averaging, EEG epochs were filtered at 25 Hz with a Tukey or cosine taper to 35 Hz, above which frequency no signal was passed. For the target stimuli waveforms, the peak (amplitude and latency) of the frontal P300 was identified at Fz and of the parietal P300 at Pz. Amplitudes were defined relative to a pre-stimulus baseline average of -300 to 0 ms. The peaks were scored within a pre-determined latency window of 220–550 ms.

### Experimental paradigm

The oddball paradigm (Figure 1) consisted of a quasi-random sequence of 280 frequent background tones (500 Hz) and 60 infrequent target tones (1000 Hz). Two targets could not appear consecutively. All stimuli (50 ms; 5 ms rise and fall time) were presented binaurally (via headphones) at a volume of 75dB SPL with an inter-stimulus interval of 1000 ms. Participants were instructed to press two buttons simultaneously (one for each index finger) when they heard a target tone and to ignore the background tones. Speed and accuracy of response were both equally stressed in the instructions. Before the actual test they were presented with a brief practice run to clarify the distinction between the two tones.

### Statistical analysis

Using Graphpad Prism 6.0, log Gaussian curves were fitted separately to amplitudes and (inverted) latencies of both the frontal and the parietal P300 across the lifespan. Curves are described by three parameters; a center value, a width value and an amplitude value. Center is the *x* value at the peak of the distribution; width is a measure of the width of the distribution expressed in the same units as *x;* amplitude is the height of the center of the distribution expressed in *y* units (www.graphpad.com). To avoid confusion the amplitude parameter will be referred to as the “height” of the model. The parameters of developmental trajectory models for the frontal and parietal P300 were statistically compared against a global model (one model explaining all data) by an extra sum-of-squares *F*-test available in Graphpad Prism 6.0. The extra-sum-of-squares *F*-test expresses how well one model fits the data vs. another simpler case of the model (i.e., the global model) by comparing the difference in sum-of-squares between the models vs. the sum-of-squares that can be expected by chance. The null hypothesis in this case is that the simpler global model is correct.

Since CRUNCH is aimed at older subjects, age-effects on (1) psychophysiological measures, i.e., P300 amplitudes and latencies, and (2) behavioral performance were evaluated in a sub-group as well. *Behavioral performance* was defined as the reaction time divided by the accuracy rate (percentage of correct responses). Notice that larger values on this scale indicate a lower performance. The sub-group consisted of all participants older than 21 years. This cut-off was based on the age at which the parietal P300 reached its maximum amplitude and compensatory frontal activity is expected to start emerging. For the frontal P300 amplitude, P300 latencies (frontal and parietal) and behavioral performance an exponential growth model was tested; for the parietal P300 amplitude an exponential decay model was tested. These models were compared against a constrained linear model representing no age-related change (slope was constrained to 0) (See the Supplementary material for the model equations and www.graphpad.com/guides/prism/6/curve-fitting for a description of the models).

Next, another measure was evaluated: The *combined P300 amplitude*, defined as the mean of the amplitudes of the frontal and parietal P300 expressing the net recruited potential, was modeled by fitting a log Gaussian curve. This model's parameters (center and width) and the parameters of a log Gaussian model for behavioral performance were statistically compared against a global model by an extra sum-of-squares *F*-test. The correlation between combined P300 amplitude and behavioral performance was calculated *post-hoc*.

Because each time two aging models were compared on three parameters a Bonferroni corrected alpha of 0.0017 (initial alpha level of 0.01, six tests) was employed.

## Results

### Developmental trajectories across the lifespan

**Figure 2:**
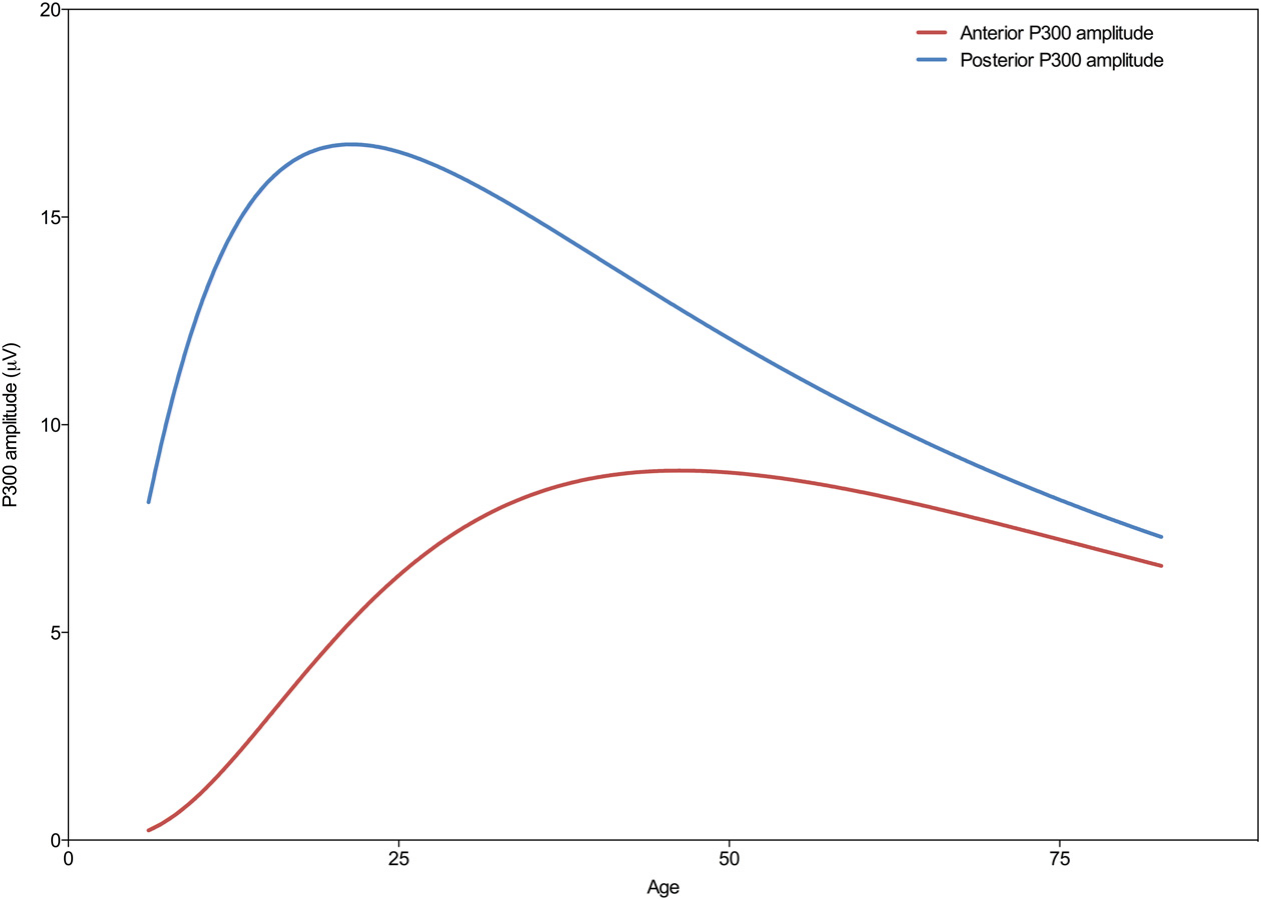
**Developmental trajectories of the frontal (Fz) and parietal (Pz) P300 amplitude across the lifespan**. Note the different trajectories of the frontal and parietal P300 amplitude across the lifespan, where the parietal P300 peaks around the age of 21 years and then shows a progressive decrease, and the frontal P300 peaks around the age of 46 years, showing a less pronounced decrease in amplitude with increasing age.

As can be seen in Figure 2 the developmental trajectories of the amplitudes for the frontal and parietal P300 are markedly different. The parietal P300 amplitude increases rapidly during childhood until a maximum is reached, after which it gradually decreases. In contrast, the amplitude of the frontal P300 reaches a plateau much later (at an age of 46 years) and decreases minimally. Statistical comparison of the curve fits for both P300 amplitudes reveals that the trajectories are significantly different [*F*_(3, 3081)_ = 583.4; *p* < 0.0001]. A *post-hoc* analysis of the separate model parameters (i.e., the center, width, and height) shows that the models differ significantly on all of these parameters, see Table 2.

**Table 2:** **Frontal vs. parietal model parameters**.

	**Center (years)**	**Width (years)**	**Height (μV/ms)**
**AMPLITUDE**			
Frontal	46.2	0.8	8.9
Parietal	21.4	1.0	16.8
*p*	<0.0001	0.0005	<0.0001
**LATENCY**			
Frontal	26.0	1.7	334.8
Parietal	24.7	1.7	342.0
*p*	0.041	0.6	0.0004

**Figure 3:**
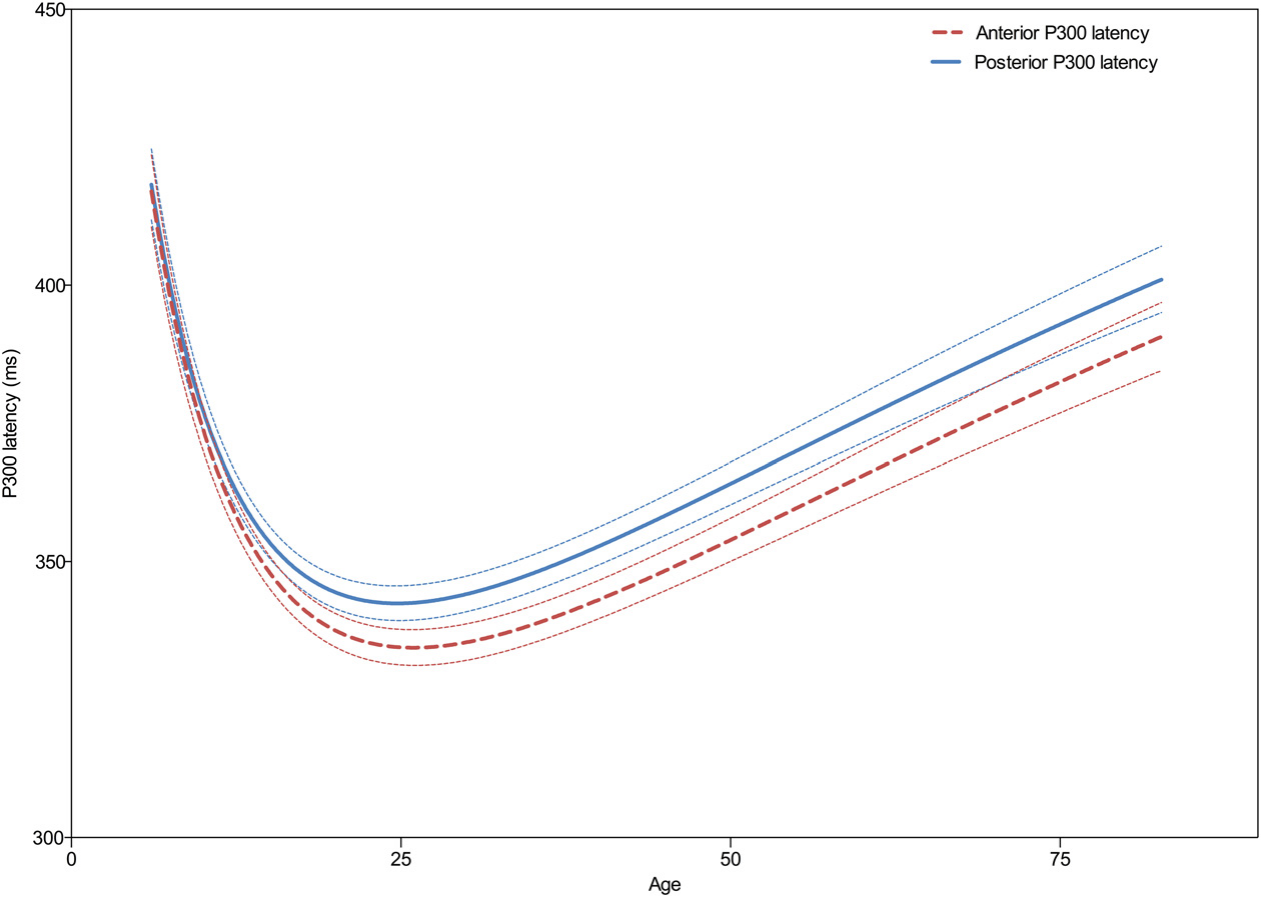
**Developmental trajectories of the frontal (Fz) and parietal (Pz) P300 latency across the lifespan**. Dashed lines indicate the 95% confidence interval. Note the similarity in developmental trajectories, with only an apparent overall difference in frontal and parietal latencies but not in its progression across the lifespan.

As can be seen in Figure 3 the developmental trajectories of the latencies for the frontal and parietal P300 are parallel. Both show a decrease in latency during childhood until a minimum is reached with a center around 25 years of age, after which latency gradually increases with aging. Statistical comparison of both models reveals that the models are significantly different [*F*_(3, 3081)_ = 11.0; *p* < 0.0001], however, this difference is driven solely by the height of the models, see Table 2. Thus, the latency of the frontal P300 is overall faster than the latency of the parietal P300. The trajectories in which they both develop across the lifespan are, in fact, very similar [When height is not included in the analysis: *F*_(2, 3081)_ = 2.2; *p* = 0.115].

### Effects of aging on psychophysiological and behavioral measures

Effects of aging on psychophysiological and behavioral measures were investigated in participants aged 21–80 years. In this sub-group, non-linear models were compared to a linear model with a slope constrained to a value of 0 (i.e., a horizontal line).

For the amplitude of the frontal P300, the exponential growth model did not significantly differ from the constrained linear model [*F*_(1, 686)_ = 4.7; *p* = 0.0313]. *Post-hoc*, an unconstrained linear model was compared to the constrained linear model to test whether amplitudes might actually be declining with aging. This analysis also demonstrated no significant difference [*F*_(1, 686)_ = 5.2; *p* = 0.0226]. For the amplitude of the parietal P300, the exponential decay model explained the data significantly better than the constrained linear model did [*F*_(2, 710)_ = 38.8; *p* < 0.0001; *R*^2^ = 0.10], suggesting an exponential decline of P300 amplitude with increasing age. Both latencies of the frontal and parietal P300 were significantly better explained by an exponential growth model than by a constrained linear model [frontal: *F*_(1, 686)_ = 139.3; *p* < 0.0001; *R*^2^ = 0.17; parietal: *F*_(1, 711)_ = 183.0; *p* < 0.0001; *R*^2^ = 0.20], indicating that P300 latencies increase with aging (exponentially). For behavioral performance an exponential growth model did not statistically differ from the constrained linear model [*F*_(1, 711)_ = 2.393; *p* = 0.122] suggesting that behavioral performance remains stable across time after 21 years of age.

**Figure 4:**
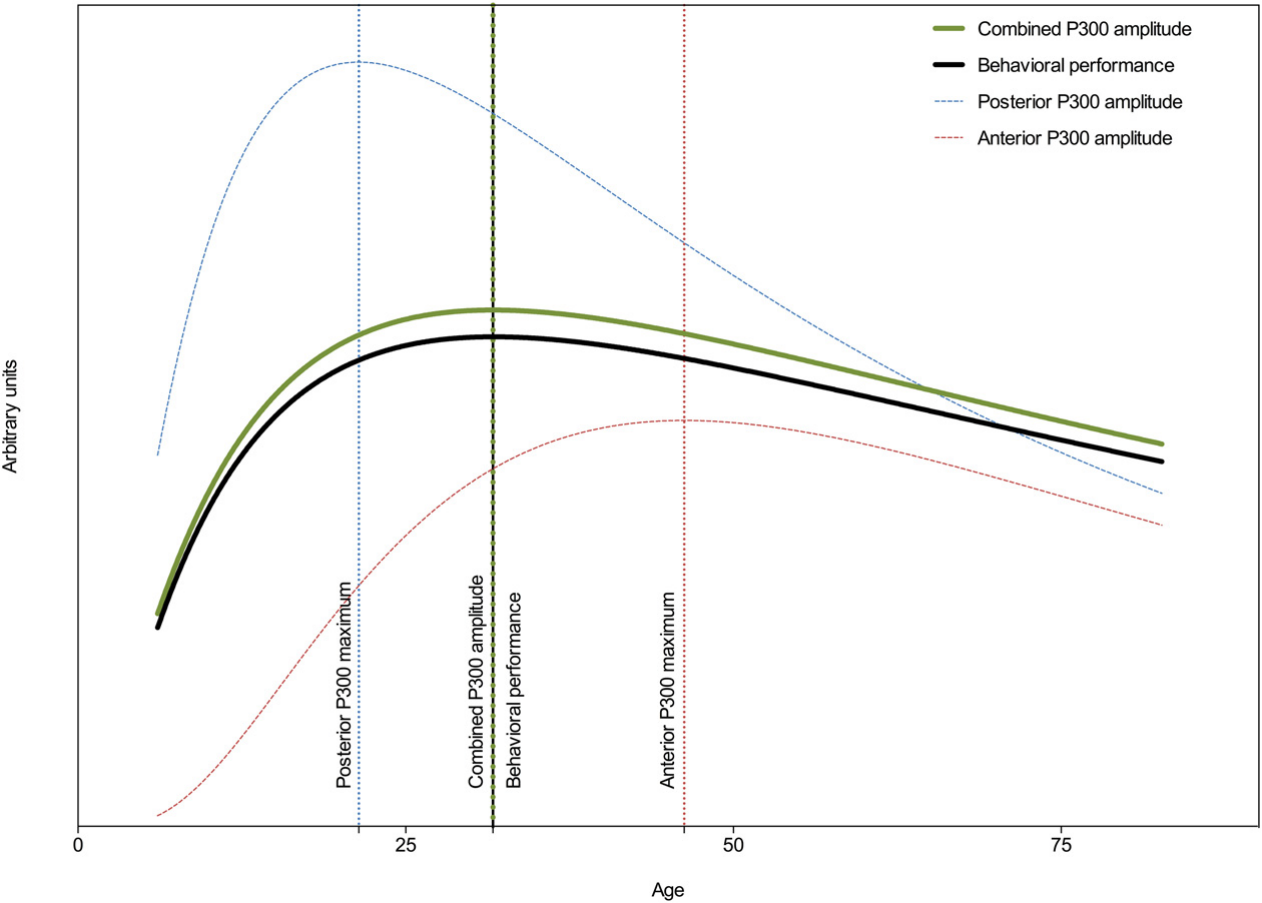
**Developmental trajectories of behavioral performance, the frontal (Fz), the parietal (Pz), and the combined P300 amplitude across the lifespan**. Vertical lines indicate points of deflection in these trajectories. Behavioral performance is plotted on an inverted y scale. Note the similarity between the developmental trajectories of the combined P300 amplitude and behavioral performance, suggesting that behavioral performance is best associated with frontal and parietal networks.

Figure 4 shows (besides the amplitude trajectories of the frontal and parietal P300 also) the combined P300 amplitude and behavioral performance across aging. An extra sum-of-squares *F*-test of the combined P300 amplitude and behavioral performance models' parameters revealed that their centers and widths were not statistically different [*F*_(2, 3081)_ = 0.03; *p* = 0.97] and a global model was the best fit. The global center was estimated at 31 years. A *post-hoc* test revealed a significant correlation between combined P300 amplitude and behavioral performance (*r* = −0.30; *p* < 0.0001; *R*^2^ = 0.09, adjusted for age).

**Figure 5:**
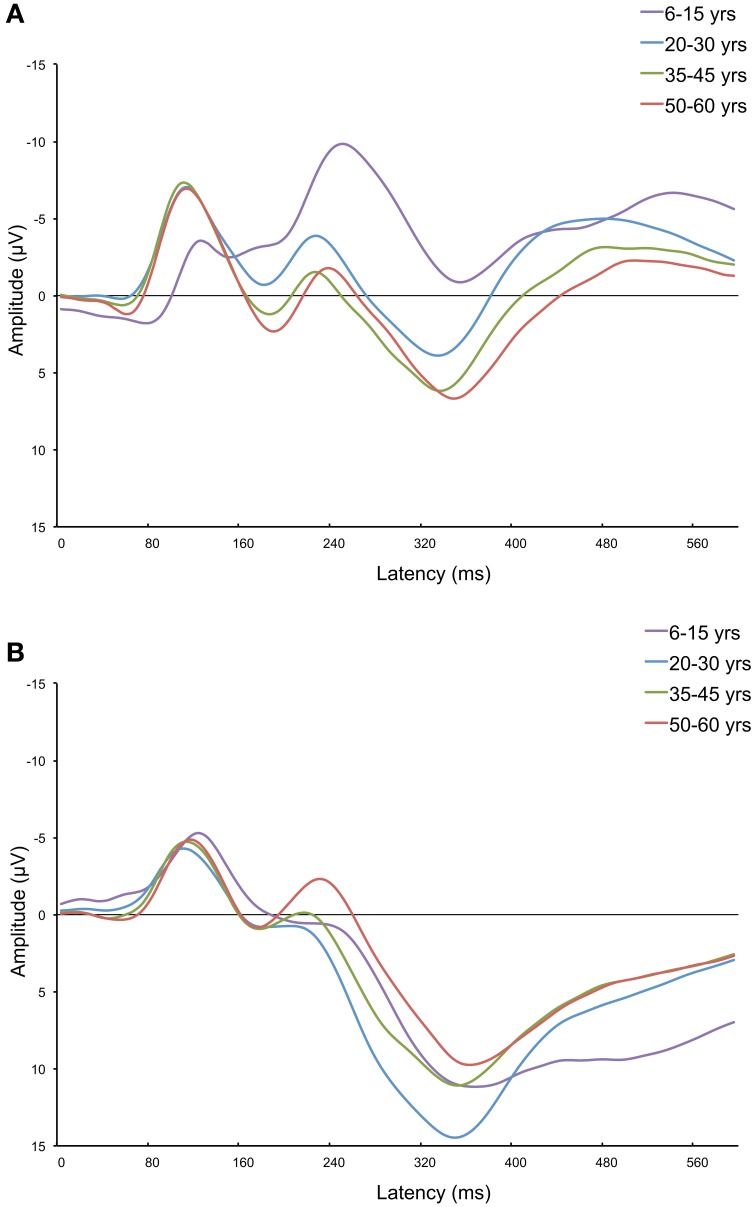
**Frontal (Fz; A) and parietal (Pz; B) grand average ERPs of four age groups: 6–15, 20–30, 35–45, and 50–60 years old**. This figure further visualizes the differences in how the frontal and parietal P300 are affected by increasing age. The frontal P300 amplitude increases with age, and the parietal P300 amplitude develops in a curvilinear way with age (an increase for children and a decrease for adults). Also note parallel changes on the N200 component (not investigated in this study).

Figures 5A,B show the grand average ERPs of four age groups (i.e., 6–15, 20–30, 35–45, and 50–60 years old) measured at Fz and Pz. These figures further demonstrate that aging has different effects on frontal vs. parietal ERPs in adults. The frontal P300 amplitude and latency increase with age. As can be seen in Figure 5B, aging has a different effect on the posterior ERP. The P300 latency increases, but its amplitude decreases (in adults). It's noteworthy that it looks like the effects are not restricted to just the P300. Other components, like the P2 (only anterior) and N2, also appear to change with aging.

## Discussion

The aim of the current study was to test CRUNCH by comparing the developmental trajectories of the frontal and parietal P300 elicited in an auditory oddball paradigm. It was expected that the developmental trajectories of the frontal and parietal P300 amplitudes would be markedly different, and that the combined frontal and parietal P300 would be better related to behavior as compared to either of these in isolation. No differences between developmental trajectories of the frontal and parietal P300 latencies were expected.

As hypothesized, trajectories of the amplitudes of the frontal and parietal P300 differed significantly. While we already demonstrated (van Dinteren et al., 2014) that the amplitude of the parietal P300 declined with aging using this sample, we here show that the amplitude of the frontal P300 did not increase with age, as would be expected from CRUNCH. Also, the frontal P300 did not *decrease* with age, as could be expected as a result of overall age-related prefrontal dysfunction. Possibly, these antagonistic effects on the frontal P300 might have canceled each other out resulting in stable amplitudes across the lifespan. However, despite the results from the lifespan analysis, an inspection of the grand average anterior ERPs of three adult age groups indicates that frontal P300 amplitudes might subtly increase with age. The significant difference between the developmental trajectories of the frontal and parietal P300 amplitudes indicates that the frontal P300 consists of other neural sources than the parietal P300 (Mulert et al., 2004; Linden, 2005; Onton et al., 2006), further confirmed by the significant difference in latency for frontal and parietal P300, which rules out volume conduction. This difference can be understood in terms of reciprocal compensation. The combined trajectory was found to be similar to the trajectory of the behavioral performance, suggesting it may reflect compensatory cognitive processes. Additionally, the correlation between the combined P300 amplitude and behavioral performance was highly significant and explained 9% of the variance in behavioral performance. Possibly, this relatively small correlation may have been due to increased variability in behavioral speed measures, such as reaction times, across the lifespan. This variability has been attributed to response-execution processes (and possibly response selection processes), but not to stimulus classification (Fjell et al., 2009), and may have reduced the correlation, especially at older ages. Although latencies of the frontal P300 were in general shorter than latencies of the parietal P300, their lifespan trajectories were similar.

The results from this study are partly in line with the CRUNCH hypothesis of Reuter-Lorenz and Cappell (2008). With aging, parietal neural resources diminish and compensatory frontal mechanisms compensate for this decline, resulting in stable behavioral performance. As people grow older neuroplastic processes may result in increased, decreased, or redistributed neural resources, and compensatory cognitive mechanisms may be required to optimize behavioral performance. As a result, it is complicated to relate the P300 amplitude to specific cognitive processes. The extent to which it reflects specific cognitive functions may also vary depending on age. That is, older people might rely more on processes reflected by the frontal P300 to compensate for a loss in resources in neural networks that the parietal P300 reflects. Behavioral performance declines only when task demands require an amount of neural resources that exceeds the maximal resources older adults can achieve with neural compensation. Thus, in order to use the P300 as an index of cognitive decline not only the parietal P300 amplitude should be evaluated, but also the frontal P300 amplitude. Adopting a lifespan approach in examining the P300 provides a unique perspective on understanding age-related changes in the brain. As was found in this study, the combination of both frontal and parietal P300 might be a good neural correlate of cognitive performance across the lifespan.

The observed results can be alternatively explained as the frontal P300 reflecting inefficient cognitive processing. As Grady (2012) stated, any (positive or negative) relation between aging and brain activity in frontal regions can be explained as compensation (successful, unsuccessful/attempted). In order to substantiate the observed results, source analysis of the P300s at frontal and parietal regions is required. Here, different underlying sources for both of these measures are hypothesized. Another explanation for the different trajectory of the amplitude from the frontal P300 compared to that of the parietal P300 could be found in the P3a vs. P3b. Unfortunately, the design of the present study does not allow for a reliable quantification of the P3a, but it would be important to investigate the developmental trajectory of its amplitude across the lifespan in future studies, especially at older ages. Possibly, the P3a plays a significant role in altering the developmental trajectory of the frontal P300 that we did not observe in the present study.

There are some limitations to this study. First, the analysis consisted of comparing age-related trajectories of P300 amplitudes and behavioral performance (cross-sectional analysis). The fact that the age-related trajectories are similar does not necessarily mean they are causally related, since for that longitudinal studies would be required. Second, we did not manipulate task load in the oddball task. It would be interesting to compare P300 amplitude trajectories for different task loads to investigate frontal compensation more directly. Third, the sample in this study was also used in our previous study (van Dinteren et al., 2014) and the obtained results should be replicated in an independent sample. However, the sample size ensures that the obtained results are reliable. Fourth, the reported P300 amplitudes were measured by a peak-picking method. The peak amplitude of an ERP component is a somewhat arbitrary measure that may not represent meaningful information about the component. Other methods of quantifying allocated cognitive resources have been proposed, for instance by determining the area under the curve. However, there were no task manipulations to influence underlying ERP components, and thereby the P300 peak, in the used oddball paradigm. Therefore, the peak of the P300 provides a reliable approximation of allocated cognitive resources in this study.

For the purposes of indicating cognitive aging the P300 is sufficient. However, as was shown in the grand average ERPs in Figure 5, the P300 seems not to be the only ERP component that changes with aging. Future studies concerning aging and ERPs should also include the P2 and N2. Furthermore, it would be interesting to compare the currently obtained developmental trajectories associated with healthy aging to those associated with pathological aging, such as seen in mild cognitive impairment or dementia. Aging-related deterioration in cognitive performance in such a clinical sample might be associated with a decline in parietal neural processes, lacking frontal compensatory processes, or both. The P300 amplitude is a solid, quick, and affordable measure that provides a more direct reflection of cognitive processing compared to behavioral measures, and that has the potential to capture these deficiencies.

### Conflict of interest statement

All participants in this study voluntarily gave written informed consent. Local IRB approval was obtained for all clinics. The authors declare that the research was conducted in the absence of any commercial or financial relationships that could be construed as a potential conflict of interest.
