# Supplementary material for: Combined frontal and parietal P300 amplitudes indicate compensated cognitive processing across the lifespan
Source: Front Aging Neurosci. 2014 Oct 24;6:294. doi: 10.3389/fnagi.2014.00294 (PMC4208402; doi:10.3389/fnagi.2014.00294)
Supplement: Supplementary file 1 [file DataSheet1.DOCX]

# Appendix: Model equations

The model equations that were used are listed below. Descriptions of the models can be found on the Graphpad Prism website ([www.graphpad.com/guides/prism/6/curve-fitting](http://www.graphpad.com/guides/prism/6/curve-fitting)).

**Log Gaussian model**

*Center* is the *x* value of the peak of the distribution; *Width* expresses the width of the distribution in the same units as *x*. *Height* is the peak of the distribution expressed in *y* units.

$$y=Height*exp(-0.5*\left( \frac{\ln\left( \frac{x}{Center} \right)}{Width} \right)^{2})$$

**Exponential growth model**

The *y* value when *x* is zero defines *y(0)*. The rate constant, *k*, is expressed in reciprocal of *x* values.

$$y=y\left( 0 \right)*exp(k*x)$$

**Exponential decay model**

The *y* value when *x* is zero defines *y(0)*. The rate constant, *k*, is expressed in reciprocal of *x* values. *Plateau* is the *y* value when *x* is infinite.

$$y=\left( y\left( 0 \right)-Plateau \right)*\exp\left( -K*x \right)+Plateau$$

**Constrained linear model**

$$y=0$$
